# Chemotherapy for unresectable and recurrent intramedullary glial tumours in children

**DOI:** 10.1038/sj.bjc.6690772

**Published:** 1999-11

**Authors:** V Doireau, J Grill, M Zerah, A Lellouch-Tubiana, D Couanet, P Chastagner, J C Marchal, Y Grignon, Z Chouffai, C Kalifa

**Affiliations:** Departments of Paediatrics and Radiology, Gustave Roussy Institute, 39-rue Camille Desmoulins, Villejuif cedex 94805, France; Departments of Paediatric Neurosurgery and Pathology, Necker Hospital, Paris, France; Departments of Paediatric Hematology-Oncology, Neurosurgery and Pathology, Children’s Hospital, Vandoeuvre, France; Center for Paediatric Hematology-Oncology, Casablanca, Morocco

**Keywords:** chemotherapy, child, intramedullary tumour, spinal cord tumour, glioma, astrocytoma

## Abstract

Adjuvant treatment for intramedullary tumours is based on radiotherapy. The place of chemotherapy in this setting has yet to be determined. Between May 1992 and January 1998, eight children with unresectable or recurrent intramedullary glioma were treated with the BB SFOP protocol (a 16-month chemotherapy regimen with carboplatin, procarbazine, vincristine, cyclophosphamide, etoposide and cisplatin). Six children had progressive disease following incomplete surgery and two had a post-operative relapse. Three patients had leptomeningeal dissemination at the outset of chemotherapy. Seven of the eight children responded clinically and radiologically, while one remained stable. At the end of the BB SFOP protocol four children were in radiological complete remission. After a median follow-up of 3 years from the beginning of chemotherapy, all the children but one (who died from another cause) are alive. Five patients remain progression-free, without radiotherapy, 59, 55, 40, 35 and 16 months after the beginning of chemotherapy. The efficacy of this chemotherapy in patients with intramedullary glial tumours calls for further trials in this setting, especially in young children and patients with metastases. © 1999 Cancer Research Campaign


British Journal of Cancer (1999) 81(5), 835–840
© 1999 Cancer Research Campaign
Article no. bjoc.1999.0772

### Chemotherapy for unresectable and recurrent intramedullary glial tumours in children

**V Doireau[1], J Grill[1], M Zerah[3], A Lellouch-Tubiana[4], D Couanet[2], P Chastagner[5], JC Marchal[6], Y Grignon[7], Z Chouffai[8] and C Kalifa[1] for the Brain Tumours Subcommittee of the French Society of Paediatric Oncology (SFOP)**

Departments of [1]Paediatrics and [2]Radiology, Gustave Roussy Institute, 39-rue Camille Desmoulins, 94805 Villejuif cedex, France; Departments of [3]Paediatric Neurosurgery and [4]Pathology, Necker Hospital, Paris, France; Departments of [5]Paediatric Hematology-Oncology, [6]Neurosurgery and [7]Pathology, Children's Hospital, Vandoeuvre, France; [8]Center for Paediatric Hematology-Oncology, Casablanca, Morocco

**Summary** Adjuvant treatment for intramedullary tumours is based on radiotherapy. The place of chemotherapy in this setting has yet to be determined. Between May 1992 and January 1998, eight children with unresectable or recurrent intramedullary glioma were treated with the BB SFOP protocol (a 16-month chemotherapy regimen with carboplatin, procarbazine, vincristine, cyclophosphamide, etoposide and cisplatin). Six children had progressive disease following incomplete surgery and two had a post-operative relapse. Three patients had leptomeningeal dissemination at the outset of chemotherapy. Seven of the eight children responded clinically and radiologically, while one remained stable. At the end of the BB SFOP protocol four children were in radiological complete remission. After a median follow-up of 3 years from the beginning of chemotherapy, all the children but one (who died from another cause) are alive. Five patients remain progression-free, without radiotherapy, 59, 55, 40, 35 and 16 months after the beginning of chemotherapy. The efficacy of this chemotherapy in patients with intramedullary glial tumours calls for further trials in this setting, especially in young children and patients with metastases.
© 1999 Cancer Research Campaign

**Keywords**: chemotherapy; child; intramedullary tumour; spinal cord tumour; glioma; astrocytoma

The standard primary treatment for spinal cord tumours is complete resection, but this can be difficult in patients with astrocytomas (Epstein and Constantini, 1995). If complete resection is impossible, or if the tumour is inoperable, radiation therapy is usually recommended (Lunardi et al, 1993; O'Sullivan et al, 1994). Metastatic tumours (with leptomeningeal spread) and relapses have a poor prognosis when treated with the standard approach, and may require other forms of treatment (Bell et al, 1988; Civitello et al, 1988; Chamberlain, 1995). In the case of holocord tumours and cases involving young children, radiotherapy (RT) can have very severe sequelae (spinal deformities, growth deficits, etc.) (Deutsch, 1995; Shirato et al, 1995; Innocenzi et al, 1996). In addition, spinal irradiation may induce anaplastic changes (Dirks, 1994) or a second malignancy, as reported in large series of spinal cord tumours (O'Sullivan et al, 1994).

Chemotherapy, mainly used for malignant intramedullary tumours in combination with radiation therapy (Allen et al, 1998), has given conflicting results. Because responses to various chemotherapy regimens have also been reported in children with low-grade gliomas (Packer et al, 1988, 1993; Petronio et al, 1991; Pons et al, 1992; Chamberlain 1995; Kalifa et al, 1995), we decided to explore the impact of chemotherapy in the management of primary intramedullary glial tumours whatever their grade. The goal was to improve survival in children with poor-prognosis tumours by the adjunction of chemotherapy, and to postpone or reduce radiotherapy in young children. Since 1992, eight children have been treated in the French BB SFOP protocol and are the focus of this report.

*Received 22 October 1998*
*Revised 10 May 1999*
*Accepted 7 June 1999*

*Correspondence to:* J Grill

### MATERIALS AND METHODS

### Eligibility

The BB SFOP protocol was initially designed for malignant central nervous system (CNS) tumours and optic pathway gliomas in children under 5. In the course of the study, patients with intramedullary tumours were enrolled if they were in relapse or had an unresectable tumour, whatever their age. Patients with a metastatic tumour at diagnosis or at relapse were also eligible. Patients with spinal cord ependymomas were ineligible. All the patients had progressive tumours and measurable disease at the start of chemotherapy. None had received radiotherapy or chemotherapy before entry to the study.

### Chemotherapy

All the patients received the same BB SFOP protocol, which is the national protocol of the French Society of Paediatric Oncology (SFOP) (Kalifa et al, 1995). It is a 16-month polychemotherapy regimen that includes seven cycles of carboplatin (15 mg $kg^{-1}$ or 450 mg $m^{-2}$ on day (d) 1), procarbazine (4 mg $kg^{-1}$ or 200 mg $m^{-2}$ on d 1–7), etoposide (5 mg $kg^{-1}$ or 150 mg $m^{-2}$ on d 22 and 23), cisplatin 1 mg $kg^{-1}$ or 30 mg $m^{-2}$ on d 22 and 23), vincristine (0.05 mg $kg^{-1}$ or 1.5 mg $m^{-2}$ on d 43) and cyclophosphamide (50 mg $kg^{-1}$ or 1500 mg $m^{-2}$ on d 43). For children over 3, doses were calculated in mg $m^{-2}$ per day.

**Table 1** McCormick functional scale

| |
|---|
| Grade I |
| Neurologically normal; mild focal deficit not significantly affecting function of involved limb; mild spasticity or reflex abnormality; normal gait. |
| Grade II |
| Presence of sensorimotor deficit affecting function of involved limb; mild to moderate gait difficulty; severe pain or dysaesthetic syndrome impairing patient's quality of life; still functions and ambulates independently. |
| Grade III |
| More severe neurological deficit; requires cane/brace for ambulation, or significant bilateral upper extremity impairment; may or may not function independently. |
| Grade IV |
| Severe deficit; requires wheelchair or cane/brace with bilateral upper extremity impairment; usually not independent. |

Reproduced with permission from McCormick et al (1990) *J Neurosurg* **72**: 523–532.

### Clinical and radiological evaluation

Clinical status was rated using the McCormick Scale (McCormick et al, 1990). This functional grading system analyses motor and sensory deficits. It includes the presence or absence of pain, without rating it (Table 1). All patients were monitored by serial magnetic resonance imaging (MRI). Films were reviewed centrally by a neuroradiologist of the SFOP expert panel (DC). Responses were assessed according to the criteria of the International Society of Paediatric Oncology (SIOP) (Gnekow, 1995).

### Histology

The slides were reviewed centrally by a neuropathologist of the SFOP expert panel (ALT). Tumours were classified according to the WHO classification (Kleihues et al, 1993).

### RESULTS

Between May 1992 and January 1998, eight children aged from 3 months to 10 years 11 months (median 4 years 11 months) were treated for primary intramedullary tumours with the BB SFOP Protocol.

### Demographic and histological data

The histological diagnoses are given in Table 2. Six of the eight patients had low-grade tumours, while two had grade III tumours (patients 1 and 8). Patients 2 and 7 did not have a second biopsy at relapse before inclusion in the study.

### Disease status before chemotherapy (Table 2)

All the tumours were progressive and three patients had metastases when chemotherapy began. The extent of the disease at the outset of chemotherapy is shown in Figure 1. Patient 1 was treated in the protocol because of a holocord neonatal tumour immediately after biopsy, and patient 4 was treated in the protocol immediately after diagnosis because he had initial metastatic disease with multiple intramedullary lesions.

### Tolerance of the chemotherapy

The first line of treatment was always surgery (biopsy in patients 1 and 4, plus a ventriculo-peritoneal shunt in patient 4; partial excision in the other six patients). Chemotherapy was started either following surgery (six patients), or at the time of relapse (32 and 45 months later, patients 7 and 2), after insertion of an implantable port, and was very well tolerated. Only three patients had blood or platelet transfusions. Five patients had six documented, easily treatable infections (*Staphyloccus epidermidis* septicaemia in three, urinary tract infections in two and varicella in one). All completed the 16-month chemotherapy regimen.

### Response to the BBSFOP protocol

Table 3 shows clinical status at the start and end of chemotherapy and at the last follow-up visit. The first symptom to disappear was pain, usually during the first cycle of chemotherapy (i.e. three courses). Seven patients had a clinical response at the end of chemotherapy (improvement of at least one grade on the McCormick functional scale), while one patient was unchanged. The clinical response was apparent after one to eight courses (median 3.5 courses). Four children had clinical signs of bladder dysfunction at the start of treatment, but they resolved during chemotherapy. At the last follow-up, none of the children had bladder dysfunction requiring urinary catheterization, although two of them remain paraplegic. Serial urodynamic results are not available for all the patients.

Table 4 shows the radiological response to BB SFOP chemotherapy. At diagnosis, all the tumours involved at least seven spinal segments, including the tumour cyst. Figure 1 shows the extent of the disease at start of chemotherapy. All the children had a measurable tumour at the outset of the BB SFOP protocol. The response to chemotherapy analyses the evolution of the tumour present at the outset.

At the end of the 16-month polychemotherapy regimen, four patients had a complete response (CR, patients 1, 5, 6 and 8). Three patients had a partial response (PR, patients 3, 4 and 7) and one had stable disease (SD, patient 2). The radiological response did not strictly match the clinical response. Patients 3 and 4 had a measurable residue after chemotherapy, even though they were totally asymptomatic. In addition, patient 1, who was in CR after chemotherapy for a holocord neonatal tumour, remains paraplegic.

Figure 2A and 2B show the radiological findings in patient 1 (holocord tumour) at the beginning and end of chemotherapy. This child entered CR during BB SFOP chemotherapy.

### Relapses after BB SFOP chemotherapy

None of the eight children had tumour progression during BB SFOP chemotherapy. Four patients relapsed or had disease progression, 1–15 months after the end of chemotherapy. Three had local and metastatic relapses (patients 2, 7 and 8) and one had only a local relapse (patient 1).

 *© 1999 Cancer Research Campaign*

**Table 2** Characteristics of the patients

| Patient | Age | Surgery | Histology | Outcome before BB SFOP |
|---|---|---|---|---|
| 1 | 3 months | Biopsy | AOA | NA (holocord tumour) |
| 2 | 11 years | Partial | OA | Local and metastatic relapse at 45 months |
| 3 | 12 months | Partial | ANOS | Growing residue at 2 months |
| 4 | 4.5 years | Biopsy + VPS | OA | NA (initial metastases) |
| 5 | 18 months | Partial | JPA | Growing residue at 2 months |
| 6 | 3 years | Partial | JPA | Growing residue at 2 months |
| 7 | 9.5 years | Partial | OA | Local and metastatic relapse at 32 months |
| 8 | 6 years | Partial | AA | Growing residue at 2 months |

VPS=ventriculoperitoneal shunt; AOA=anaplastic oligo-astrocytoma; OA=oligoastrocytoma; ANOS=astrocytoma not otherwise specified; JPA=juvenile pilocytic astrocytoma; AA=anaplastic astrocytoma; NA=not available because the chemotherapy was initiated shortly after diagnosis because of the status of the disease.

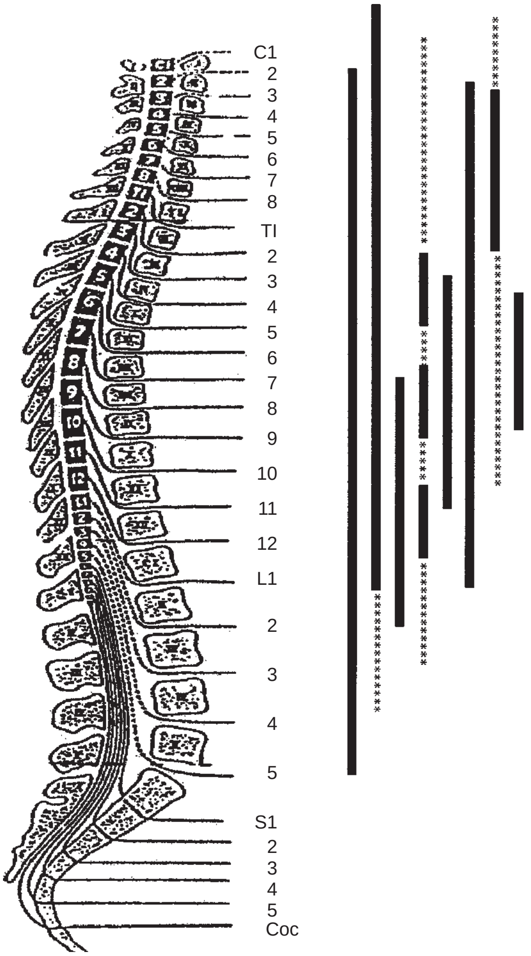

**Figure 1** Disease extension at the beginning of chemotherapy. The extension of the disease along the spinal cord is given, and includes the cyst. The order of the bars from left to right follow the order of the patients in the study (patient 1 on the left). The asterisks indicate patients with leptomeningeal spread

Second-line treatment was a monthly vincristine–carboplatin combination in three according to the protocol of the International Society of Paediatric Oncology (SIOP) (Perilongo, 1998), adapted from the regimen published by Packer et al (1997). The responses were PR in one (patient 1), PD in one (patient 7) and unevaluable in one (patient 8, who died in a car accident). Patient 1 was operated on for a small spinal residue after 1 year of treatment with the vincristine–carboplatin combination. Histological analysis showed only mature glial cells. This child is alive and disease-free 1.5 years after the end of treatment.

**Table 3** Clinical outcome during and after chemotherapy according to the McCormick scale

| Patient | Age at diagnosis | Start | End | Last visit | Follow-up |
|---|---|---|---|---|---|
| 1 | 3 months | IV | III | III | 5.5 years |
| 2 | 11 years | III | III | II* | 3 years |
| 3 | 1 year | III | I | I | 5.5 years |
| 4 | 4.5 years | III | II | I | 4 years |
| 5 | 1.5 years | IV | I | I | 3.5 years |
| 6 | 3 years | III | I | I | 3 years |
| 7 | 9.5 years | III | I | III* | 3 years |
| 8 | 6 years | IV | I | NE* | DWD |

NE = not evaluable because of death from other causes. * = relapse after completion of BBSFOP protocol. DWD = death with disease. Follow-up duration is given from beginning of chemotherapy.

Patient 7 had a partial response to third-line chemotherapy consisting of low-dose oral etoposide (Chamberlain, 1995) and was subsequently irradiated (35 Gy) to the whole CNS with a further improvement. He was alive and still had disease 3 months after the end of radiotherapy.

Patient 2 was irradiated at 35 Gy to the whole CNS as second-line treatment at the time of relapse. Craniospinal irradiation yielded a major clinical improvement that persisted after 12 months of follow-up.

Three of the four patients who relapsed after the BB SFOP protocol were still alive, 59, 63 and 71 months after initial diagnosis.

### Survival

In August 1998, the median follow-up was 4.8 years (1.8–5.8 years) after first treatment, and 3.8 years (1.8–5.3 years) after the beginning of chemotherapy. Seven patients are alive, while the last died in a car accident. The overall survival rate is 87.5% and the event-free survival rate at 2 and 4 years is 50%. At the last follow-up visit five patients (patients 2, 3, 4, 5 and 6) were ambulatory (3–5.5 years after beginning of chemotherapy). Two patients are paraplegic; one was quadriplegic at diagnosis and one became paraplegic after a second relapse. Four patients are in continuous remission without the use of radiation therapy (patients 1, 4, 5 and 6).

© 1999 Cancer Research Campaign

**Table 4** Radiological response during and after chemotherapy

| Patient | Histology | Tumour | Response at end of BBSFOP | Status of disease at follow-up |
|---|---|---|---|---|
| 1 | AOA | Holocord, not operated | CR | CR2 at 5.5 years[a] |
| 2 | OA | Local and metastatic relapse | SD | PR[b] at 3 years |
| 3 | ANOS | Residue | PR | SD at 5.5 years |
| 4 | OA | Initial metastases, not operated | PR | VGPR at 4 years |
| 5 | JPA | Residue | CR | CCR at 3.5 years |
| 6 | JPA | Residue | CR | CCR at 3 years |
| 7 | OA | Local and metastatic relapse | PR | SD[c] at 3 years |
| 8 | AA | Residue | CR | DWD[d] at 32 months |

The response to BB SFOP protocol is analysed by comparing the MRI performed just before chemotherapy and the one performed at the end of chemotherapy. Changes in the contrast enhancement of the lesion without modification of the size were not considered as a response. If there is discrepancy between the evolution of multiple lesions, only the worst response is considered. The status of the disease is analysed by comparing the MRI at the end of the chemotherapy and the last available radiological films. [a]One year after the end of BB SFOP chemotherapy, this patient had a local relapse that showed a partial response to a second-line chemotherapy. A complete surgical resection of the residue was performed and pathological analysis showed only mature glial cells. [b]The patient 2 was irradiated shortly after the end of chemotherapy with clinical signs of tumour progression. [c]The patient 7 relapsed again 22 months after diagnosis of his first relapse (i.e. after the start of BB SFOP chemotherapy), exhibited a partial response to a second-line chemotherapy (oral etoposide) of 6 months duration and was subsequently irradiated. [d]The patient 8 had a metastatic relapse 2.5 years after the diagnosis, received salvage treatment with second-line chemotherapy but died in a car accident 32 months from diagnosis while on treatment.

A B

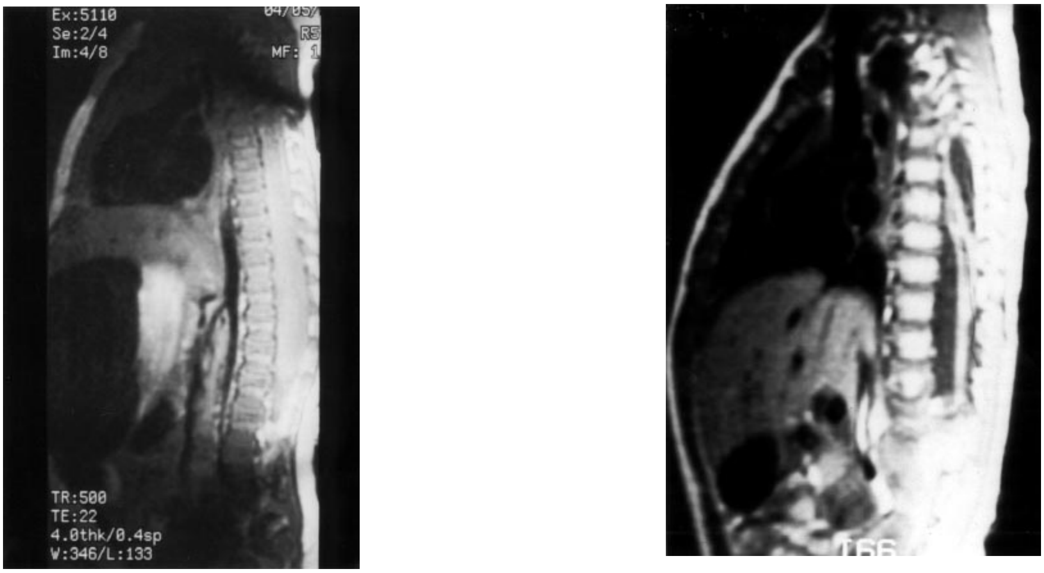

**Figure 2** Response to BB SFOP chemotherapy in a 3-month-old girl with a holocord tumour. This 3-month-old baby presented with quadriplegia, the Claude Bernard Horner sign and stiff neck. MRI showed a holocord tumour from C2 to L5 with no evidence of leptomeningeal spread (**A**). After the first cycle, her clinical condition improved dramatically, in parallel with radiological signs. At the end of BB SFOP chemotherapy she remained paraplegic without significant bladder dysfunction, and MRI showed a complete response (**B**)

### DISCUSSION

Paediatric intramedullary spinal cord tumours are rare, and so are data on treatment strategies and outcomes. Surgery alone is usually the treatment of choice for low-grade tumours, provided that subtotal resection is possible (Guidetti et al, 1981; Epstein and Epstein, 1982; Lunardi et al, 1993; Epstein and Constantini, 1995; Goh et al, 1997; Mottl and Koutecky, 1997). Adjuvant treatment with irradiation and/or chemotherapy are usually reserved for high-grade or metastatic tumours, as their prognosis with surgery alone is poor (Cohen et al, 1989; Bouffet et al, 1995; Goh et al, 1997; Allen et al, 1998). However, adjuvant treatment may be considered even for low-grade tumours after incomplete surgery or metastatic or local relapses, and it may be the first line of treatment for unresectable tumours. The place of chemotherapy in these situations has not previously been explored. We report the first series of patients treated with a single chemotherapy protocol (BB SFOP). Our results show that this treatment is safe and very effective, in clinical and radiological terms, in patients whose prognosis is otherwise poor.

There are only a few case reports of this treatment modality in intramedullary astrocytoma. Bouffet et al (1997) reported the case of a 30-year-old woman with a grade II spinal cord astrocytoma treated first with carboplatin, who entered full neurological and radiological remission. The result was promising despite the short follow-up. However, using the same chemotherapy, Foreman et al (1998) reported a treatment failure in an 8-year-old child treated at time of relapse of a low-grade glioma. In a report from Bristol (Lowis et al, 1998), responses to chemotherapy were observed in a 19-month-old child with anaplastic astrocytoma of the cervical

 *© 1999 Cancer Research Campaign*

spinal cord, and in a 4-year-old child with a recurrent low-grade astrocytoma. Again, follow-up was short in both cases.

Unpublished data on the use of vincristine–carboplatin combination in intramedullary tumours are available from the Children's Cancer Group and the SIOP consortium for low-grade tumours. Fort and Packer reported one CR and one PR in five patients at the Paediatric Neuro-Oncology Symposium in Rome (Fort et al, 1998). In the SIOP protocol, two of four assessable patients with low-grade spinal gliomas responded to this combination (Perilongo 1998).

At the outset of chemotherapy, three of our patients had leptomeningeal metastases; this is a rare event in benign CNS tumours in children (Hardison et al, 1987; Bell et al, 1988; Civitello et al, 1988), being more frequent with intramedullary tumours and malignant tumours (Chamberlain, 1995). Chemotherapy induced two PR and one SD in our three children with leptomeningeal dissemination. One of the children with PR (patient 4) is progression-free 4 years after diagnosis. The other child with PR (patient 7) and the child with SD (patient 2) subsequently relapsed, were irradiated and remain progression-free (3 months and 1 year after completion of RT respectively). Survival after leptomeningeal relapses of intramedullary tumours is poor. One of the three children reported by Bell et al (1988) was treated with systemic chemotherapy and was alive 10 months after relapsing, with stable residual disease. All the patients reported by Chamberlain in a review of the paediatric literature in 1995 (Chamberlain, 1995) died within 6 months of the onset of leptomeningeal dissemination. They had various primary tumours and were treated with RT and intrathecal chemotherapy. Two of the three patients reported by Civitello et al (1988) were alive 9 and 44 months after relapsing. They both received chemotherapy, and one also had craniospinal radiotherapy (csRT). The two children reported by Hardison et al (1987) were treated with both chemotherapy and csRT and died 10 and 11 months later. Although it is clear that dissemination is associated with very poor outcome in patients with glial tumours, it is not clear whether early and secondary dissemination of glial tumours have the same prognostic impact.

Even in our two patients with high-grade tumours we obtained lengthy complete remissions; this is quite unusual according to the literature on malignant astrocytomas, which are characterized by rapid neurological deterioration and death (Cohen et al, 1989; Allen et al, 1998).

The most interesting result of chemotherapy was a marked improvement in functional status: our results compare favourably with those of surgery in other paediatric (Epstein and Epstein, 1982; Reimer and Onofrio, 1985; Hardison et al, 1987; Constantini et al, 1996; Innocenzi et al, 1996; Goh et al, 1997) and adult series (Guidetti et al, 1981; Cooper, 1989). In the paediatric literature, clinical improvement after surgery is observed in 0.7–73% of cases (Epstein and Constantini, 1995; Goh et al, 1997). The neurosurgeons of New York University obtained improvements in 18.5% of cases after surgery of intramedullary spinal cord tumours in children under 3 years of age (Constantini et al, 1996). In this latter series, 69% of patients were ambulatory with 76 months of follow-up. Functional status improved after surgery in only 2/29 children with astrocytomas reported by Innocenzi and Raimondi, and worsened in four cases (Innocenzi et al, 1996). In an adult series with long-term results (mean 38 months) for 51 intramedeuallary spinal cord tumours after surgery, 29% patients were improved, 80% were ambulatory and 20% were neurologically intact (Cooper et al, 1989). Guidetti et al (1981) report their treatment results for 53 adults with intramedullary astrocytomas; only 10% were improved after surgical treatment (12–324 months follow-up). In our study, functional status improved even in the patient with a holocord tumour. In addition, side-effects were minor compared with those described after RT (O'Sullivan et al, 1994; Deutsch, 1995; Shirato et al, 1995) or extensive surgery (Guidetti et al, 1981; Reimer and Onofrio, 1985; Innocenzi et al, 1996).

Survival in this small series was good. None of the patients died of their disease, and half of them had no relapse after a median follow-up of 5 years from diagnosis and 4 years from the outset of chemotherapy. In the main paediatric series of intramedullary tumours (Epstein and Epstein, 1982; Reimer and Onofrio, 1985; Hardison et al, 1987; Lunardi et al, 1993; Constantini et al, 1996; Innocenzi et al, 1996; Goh et al, 1997; Mottl et al, 1997), treatment consisted of surgery alone or combined with radiotherapy. With a follow-up of 5 years, the reported overall survival rate was between 39 and 96%, and the event-free survival rate was between 14 and 77%.

In conclusion, chemotherapy should be considered for children with a poor prognosis due to relapse or metastasis of an intramedullary tumour. Chemotherapy could even be the first line of treatment for metastatic and unresectable tumours (e.g. holocord tumours) and replace extensive irradiation. Moreover, when adjuvant treatment is indicated after incomplete resection, chemotherapy may delay or avoid the use of irradiation, which can have severe sequelae in young children. Because these tumours are rare, large cooperative studies will be necessary to further explore the value of chemotherapy in intramedullary glial tumours.

### REFERENCES

Allen JC, Aviner S, Yates AJ, Boyett JM, Cherlow JM, Turski PA, Epstein F and Finlay JL (1998) Treatment of high-grade spinal cord astrocytoma of childhood with '8 in 1' chemotherapy and radiotherapy: a pilot study of CCG-945. Children's Cancer Group. *J Neurosurg* **88**: 215–220

Bell WO, Packer RJ, Seigel KR, Rorke LB, Sutton LN, Bruce DA and Schut L (1988) Leptomeningeal spread of intramedullary spinal cord tumours: report of three cases. *J Neurosurg* **69**: 295–300

Bouffet E, Pierre-Kahn A, Choux M, Marchal JC, Dhellemes P, Gurin J, Tremoulet M, Roche JL, Ravon R, Stilhart B, Chazal J, Passagia JG, Jouvet A and Mottolese C (1995) Spinal cord astrocytoma in childhood: a multicentric study of 74 cases (SIOP XXVII Meeting abstract). *Med Ped Oncol* **25**: 247

Bouffet E, Amat D, Devaux Y and Desuzinges C (1997) Chemotherapy for spinal cord astrocytoma. *Med Ped Oncol* **29**: 560–562

Chamberlain MC and Grafe MR (1995) Recurrent chiasmatic hypothalamic glioma treated with oral etoposide. *J Clin Oncol* **13**: 2072–2076

Chamberlain MC (1995) A review of leptomeningeal metastasis in pediatrics. *J Child Neurol* **10**: 91–199

Civitello LA, Packer RJ, Rorke LB et al (1988) Leptomeningeal dissemination of low-grade gliomas in childhood. *Neurology* **38**: 562–566

Cohen AR, Wisoff JH, Allen JC and Epstein F (1989) Malignant astrocytomas of the spinal cord. *J Neurosurg* **70**: 50–54

Constantini S, Houten J, Miller DC, Freed D, Ozek MM, Rorke LB, Allen JC and Epstein F (1996) Intramedullary spinal cord tumours in children under the age of 3 years. *J Neurosurg* **85**: 1036–1043

Cooper PR (1989) Outcome after operative treatment of intramedullary spinal cord tumours in adults: intermediate and long term results in 51 patients. *Neurosurgery* **25**: 855–859

Deutsch M (1995) Sequelae of spine irradiation in children. In: *Disorders of the Pediatric Spine*, D Pang (ed), pp. 421–443. Raven: New York

Dirks PB (1994) Development of anaplastic changes in low-grade astrocytomas of childhood. *Neurosurgery* **34**: 68–78

Epstein FJ and Constantini S (1995) Spinal cord tumours of childhood. In: *Disorders of the Pediatric Spine,* D Pang (ed), pp. 371–388. Raven: New York

Epstein FJ and Epstein N (1982) Surgical treatment of spinal cord astrocytomas of childhood. A series of 19 patients. *J Neurosurg* **57**: 685–689

*© 1999 Cancer Research Campaign* 

Foreman NK, Hay TC and Handler M (1998). Chemotherapy for spinal cord astrocytoma. *Med Ped Oncol* **30**: 311–312 (letter).

Fort DW, Packer RJ, Kirckpatrick GB, Kuttesch JF Jr and Ater JL (1998) Carboplatin and vincristine for pediatric spinal cord astrocytomas (8th International Neuro-Oncology Symposium abstract). *Child's Nerv Syst* **14**: 484

Gnekow A (1995) Recommendations of the brain tumour subcommittee for the reporting of trials. *Med Ped Oncol* **24**: 104–108

Goh KY, Velasquez L and Epstein FJ (1997) Pediatric intramedullary spinal cord tumours: is surgery alone enough? *Pediatr Neurosurg* **27**: 34–39

Guidetti B, Mercuri S and Vagnozzi R (1981) Long term results of the surgical treatment of 129 intramedullary spinal gliomas. *J Neurosurg* **54**: 323–330

Hardison HH, Packer RJ, Rorke LB, Schut L, Sutton LN and Bruce DA (1987) Outcome of children with primary intramedullary spinal cord tumours. *Child's Nerv Syst* **3**: 89–92

Innocenzi G, Raco A, Cantore G and Raimondi AJ (1996) Intramedullary astrocytomas and ependymomas in the pediatric age group: a retrospective study. *Child's Nerv Syst* **12**: 776–780

Kalifa C, Raquin MA, Plantaz F, Doz F, Chastagner P, Barenzelli MC, Bouffet E, Couanet D, for the French Society of Paediatric Oncology (SFOP) (1995) Chemotherapy for low grade gliomas (SIOP XXVII Meeting Abstract). *Med Pediatr Oncol* **25**: 246

Kleihues P, Burger PC and Scheithauer BW (1993) The new WHO classification of brain tumours. *Brain Pathol* **3**: 255–268

Lowis SP, Pizer BL, Coakham H, Nelson RJ and Bouffet E (1998) Chemotherapy for spinal cord astrocytoma: can natural history be modified? *Childs Nerv Syst* **14**: 317–321

Lunardi P, Licastro G, Missori P, Ferrante L and Fortuna A (1993) Management of intramedullary tumours in children. *Acta Neurochir (Wien)* **120**: 59–65

McCormick PC, Torres R, Post KD and Stein BM (1990) Intramedullary ependymoma of the spinal cord. *J Neurosurg* **72**: 523–532

Mottl H and Koutecky J (1997) Treatment of spinal cord tumours in children. *Med Ped Oncol* **29**: 293–295

O'Sullivan C, Jenkin RD, Doherty MA, Hoffman HJ and Greenberg ML (1994) Spinal cord tumours in children: long term results of combined surgical and radiation treatment. *J Neurosurg* **81**: 507–512

Packer RJ, Sutton L, Bilaniuk L, Radcliffe J, Rosenstock JG, Siegel KR, Bunin GR, Savino PJ, Bruce DA and Schut L (1988) Treatment of chiasmatic/ hypothalamic gliomas of childhood with chemotherapy. *Ann Neurol* **23**: 79–85

Packer RJ, Lange B, Ater J, Nicholson HS, Allen J, Walker R, Prados M, Jakacki R, Reaman G and Needle MN (1993) Carboplatin and Vincristine for recurrent and newly diagnosed low grade gliomas of childhood. *J Clin Oncol* **11**: 850–856

Petronio J, Coward MSB, Prados M, Freyberger S, Rabbitt J, Silver P and Levin VA (1991) Management of chiasmal and hypothalamic gliomas of infancy and childhood with chemotherapy. *J Neurosurg* **74**: 701–708

Perilongo G, Gnekow AK, Walker D, Zanetti I, Garré ML, Kühl J and Taylor R (1998) Single dose carboplatin and vincristine chemotherapy for childhood low-grade gliomas. (EANO III Meeting abstract). *J Neuro-Oncol* **39**: 105

Pons MA, Finlay JL, Walker RW, Puccetti D, Packer RJ and McElwain M (1992) Chemotherapy with Vincristine and Etoposide in children with low-grade astrocytoma. *J Neuro-Oncol* **14**: 151–158

Reimer R and Onofrio BM (1985) Astrocytomas of the spinal cord in children and adolescents. *J Neurosurg* **63**: 669–675

Shirato H, Kamada T, Hida K, Koyanagi I, Iwasaki Y, Miyasaka K and Abe H (1995) The role of radiotherapy in the management of spinal cord glioma. *Int J Radiat Oncol Biol Phys* **33**: 323–328

 *© 1999 Cancer Research Campaign*
